# The neural signatures of psychoses in Alzheimer’s disease: a neuroimaging genetics approach

**DOI:** 10.1007/s00406-022-01432-6

**Published:** 2022-06-21

**Authors:** Riccardo Manca, Antonio F. Pardiñas, Annalena Venneri, for the Alzheimer’s Disease Neuroimaging Initiative

**Affiliations:** 1 Department of Life Sciences, Brunel University London, Uxbridge, London, UK; 2 MRC Centre for Neuropsychiatric Genetics and Genomics, Division of Psychological Medicine and Clinical Neurosciences, School of Medicine, Cardiff University, Cardiff, UK; 3 Department of Medicine and Surgery, University of Parma, Parma, Italy

**Keywords:** Polygenic risk, Neuropsychiatric, Dementia, Schizophrenia, Disconnection, Orbitofrontal

## Abstract

Psychoses in Alzheimer’s disease (AD) are associated with worse prognosis. Genetic vulnerability for schizophrenia (SCZ) may drive AD-related psychoses, yet its impact on brain constituents is still unknown. This study aimed to investigate the association between polygenic risk scores (PRSs) for SCZ and psychotic experiences (PE) and grey matter (GM) volume in patients with AD with (AD-PS) and without (AD-NP) psychosis. Clinical, genetic and T1-weighted MRI data for 800 participants were extracted from the ADNI database: 203 healthy controls, 121 AD-PS and 476 AD-NP. PRSs were calculated using a Bayesian approach and analysed at ten *p*-value thresholds. Standard voxel-based morphometry was used to process MRI data. Logistic regression models including both PRSs for SCZ and PE, and an AD-PRS were used to predict psychosis in AD. Associations between PRSs and GM volume were investigated in the whole sample and the three groups independently. Only the AD-PRS predicted psychosis in AD. Inconsistent associations between the SCZ-PRS and PE-PRS and GM volumes were found across groups. The SCZ-PRS was negatively associated with medio-temporal/subcortical volumes and positively with medial/orbitofrontal volumes in the AD-PS group. Only medio-temporal areas were more atrophic in the AD-PS group, while there was no significant correlation between psychosis severity and GM volume. Although not associated with psychoses, the SCZ-PRS was correlated with smaller medio-temporal and larger orbitofrontal volumes in AD-PS. Similar alterations have also been observed in SCZ patients. This finding suggest a possible disconnection between these regions associated with psychoses in more advanced AD.

**Supplementary Information:**

The online version contains supplementary material available at 10.1007/s00406-022-01432-6.

## Introduction

Alzheimer’s disease (AD) is characterised by a heterogeneous symptomatic presentation, commonly including behavioural disturbance along with cognitive decline. About 30% of patients with AD present with psychotic symptoms, i.e. either delusions or hallucinations [1]. Both delusions and hallucinations have been found to emerge in advanced disease stages [2], although there may be differences in the temporal onset of specific psychotic symptoms [3]. Psychoses have been associated with poorer health outcomes in people with AD, such as worse cognitive decline [4], higher risk of hospitalisation and greater distress in patients and carers [5].

These detrimental effects may be partially explained by psychotic symptoms reflecting pathology-related cerebral changes in AD, including: increased concentration of hyperphosphorylated tau protein in different frontal areas [6, 7]; macrostructural and functional brain alterations across a range of areas including bilateral frontal, parietal and striatal regions [8]. Serra et al. [9] found that severity of delusions was negatively associated with the volume of the right hippocampus and middle frontal gyrus. Moreover, there is evidence that patients with AD and delusions have stronger functional connectivity in frontal regions [10] and weaker functional connectivity in the left inferior parietal lobule [11]. Hallucinations in AD have also been associated with cortical thinning in the supramarginal gyrus [12] and with smaller volume and hypometabolism in right insular, superior temporal and prefrontal areas [13]. Such variability may be due to the fact that different psychotic symptoms may be driven by partially distinct neuropathological processes and potentially by pathological changes unrelated to AD, such as Lewy bodies, vascular damage and leukoencephalopathy, that might explain psychotic symptoms in this clinical population [14].

Carriers of the Apolipoprotein E (*APOE*) ε4 allele, i.e. the strongest genetic risk factor for sporadic late-onset AD [15–17], appear at higher risk of AD-related psychosis, although this finding has not been replicated by all studies [18]. Several other genes, involved in a variety of functions but mostly linked to schizophrenia (SCZ), also appear linked to psychosis status in AD, although evidence is varied [19–22]. In fact, genome-wide association studies (GWAS) have highlighted significant associations between psychosis in AD and multiple SNPs, including the *APOE* gene [23], supporting the hypothesis that a multifaceted genetic underpinning may underlie this phenotype.

These findings altogether suggest the existence of a genetic association between SCZ and AD-related psychosis, probably driven by pleiotropic effects of multiple genes [24]. To investigate such claims, recent studies used polygenic risk scores (PRSs) for SCZ to predict psychosis in patients with AD. DeMichele-Sweet et al. [25] found that these symptoms were positively associated with a subset of trait-specific SNPs, but negatively associated with SCZ-PRS. A recent meta-analysis, however, observed that higher SCZ-PRS predicted psychosis in AD, although associations in individual cohorts were not consistent [26]. Further support for potential pleiotropic effects of SCZ-related genes comes from a recent study that found a SCZ-PRS to be associated with psychosis in Huntington’s disease [27].

Different lines of research converge to suggest that the potential biological correlates of psychotic symptoms in people with AD are complex and possibly determined by multiple factors. However, to date no studies have investigated whether genetic risks (e.g. SCZ-PRS) are associated not only with behavioural traits (e.g. psychosis) but also with brain parameters in people with AD. For this reason, the aim of this exploratory study, which is the first of its kind to the best of our knowledge, was to use data from the Alzheimer’s Disease Neuroimaging Initiative (ADNI) to: 1) test weather a PRS for SCZ and a novel PRS for psychotic experiences in the general population would be associated with risk of psychosis in patients with AD (higher PRSs were expected to be associated with higher risk); 2) investigate whether the two PRSs were differentially associated with regional grey matter (GM) volume in two groups of patients with AD with and without psychosis.

## Methods and materials

### Participants

A sample of 812 individuals with genotyping data were initially screened for inclusion. Data used in the preparation of this article were obtained from the Alzheimer’s Disease Neuroimaging Initiative (ADNI) database (adni.loni.usc.edu).^1^ Institutional review boards of each site involved in ADNI approved the study protocol and all participants provided written informed consent. Approval for secondary analyses of this dataset was granted by the Research Committee of Brunel University London (reference number 30422-TISS-Jul/2021- 33,453–2). Participants were included on the basis of the availability of genetic, MRI, neuropsychiatric and cognitive assessments. The lack of any of the abovementioned assessments and a history of previous chronic psychiatric diagnosis represented exclusion criteria for this study.

In the initial sample 601 participants received a diagnosis of either MCI or dementia due to AD, while 211 participants were healthy controls (HC), since no evidence of cognitive decline was detected longitudinally at any of the available follow-up time points. Two groups of patients were identified: 121 presenting with psychosis (AD-PS), either delusions or hallucinations recorded by means of either the Neuropsychiatric Inventory (NPI) [28] or the NPI-Questionnaire [29], and 480 with no evidence of psychosis at any time-point (AD-NP). Six HC were also found to present with psychosis and, therefore, were discarded due to concerns about the presence of potential late-onset psychosis unrelated to AD pathology. Subsequently, 5 couples of siblings were identified, hence one participant per couple was removed from our analysis to avoid biases due to relatedness [30]. Finally, one patient without psychosis was removed because no MRI data were available. The final sample consisted of a total of 800 participants: 203 HC, 476 AD-NP (383 with MCI and 83 with dementia) and 121 AD-PS (49 with MCI and 72 with dementia). To maximise our sample size and potential transferability of results [31], all participants were retained, including 6.5% from a minority ethnic background (52 non-white out of 800), although there was no significant difference in ethnicity distribution across groups (χ^2^ = 9.60, *p* = 0.65).

### Clinical and cognitive data

Severity of neuropsychiatric symptoms was assessed by means of the NPI/NPI-Q, according to availability. Differently from the NPI-Q, the NPI assessment provides a total score that combines information about severity and frequency of symptoms. To quantify neuropsychiatric symptoms homogenously across participants, all NPI scores were converted into NPI-Q-like scores.

To characterise the cognitive profile of the sample, scores on a series of cognitive tests collected at the time-point closest to the neuropsychiatric assessment were also extracted. The following tests were included: Mini Mental State Examination (MMSE) [32], Clock Drawing Test, both free drawing and copy [33], Logical Memory Test, both immediate and delayed recall [34], Category Fluency Test—animals [35] and completion time of part A of the Trail Making Test [36].

### Genetic data and polygenic risk scores

Apolipoprotein E (*APOE*) genotype status for all participants was available in the ADNI database. Genotyping was carried out by ADNI using an Illumina OmniExpress array [37]. Genotype data were curated to extract common high-quality autosomal markers using PLINKv2.0 [38]. Quality control parameters were 90% call rate, 5% minor allele frequency and Hardy–Weinberg equilibrium mid-p value 10^–6^. A total of 1.3 million single nucleotide polymorphisms (SNPs) passed quality control. From the quality-controlled genotype data, genetic principal components (PCs) were generated using PC-AiR [39] and the first 10 were used as covariates for regression analyses. Robust relatedness estimates, corrected for 3 PCs, were generated using PC-Relate [40]. Both approaches are implemented in the “GENESIS” R package [41].

Three polygenic risk scores (PRS) were calculated for each participant using different training sets and GWAS summary statistics: a schizophrenia PRS (SCZ-PRS) [42], one for psychotic experiences in the general population (PE-PRS) [43] and one for AD (AD-PRS) [44]. Only SNPs with imputation information content (INFO) scores greater than 0.9 were used and duplicate SNPs were removed. To our knowledge, participants in ADNI were not included in any of the discovery GWAS used to calculate PRSs.

The PE-PRS was investigated as an index complementary to the SCZ-PRS since the limited literature in this field has highlighted contrasting results [25, 26] and the development of psychoses in psychiatric disorders (and, sporadically, among the general non-psychiatric population) appears to have a different genetic basis [43]. The AD-PRS, instead, was included since AD-related risk factors have been found to contribute also to psychosis in this population [15–17].

PRSs were generated using a Bayesian approach using continuous shrinkage priors (PRS-CS) [45]. After merging the data with a linkage disequilibrium reference based on the 1000 Genomes EUR samples, 334,976 SNPs were retained for SCZ, 446,852 SNPs for PE and 455,027 for AD. From each of those SNPs a Bayesian posterior effect size was calculated. The shrinkage parameter was set to φ = 1 for SCZ [46] and inferred using PRS-CS-auto for PE and AD. Finally, posterior effect sizes were used to calculate PRSs in PRSice v2 [47] without pruning at 10 GWAS *p* value thresholds (*P*_T_): 5 × 10^–8^, 1 × 10^–6^, 1 × 10^–5^, 0.001, 0.001, 0.01, 0.05, 0.1, 0.5 and 1. However, no SNPs were retained during the calculation of the PE-PRS at the two most conservative P_T_ (5 × 10^–8^ and 1 × 10^–6^) due to the more modest power of this GWAS. PRSs were standardised (centring by mean and dividing by one standard deviation) to be used in the analysis.

PRS scores were then used to predict psychosis status in patients (AD-PS vs AD-NP) in logistic regression models including the following predictors: the psychiatric PRS (either SCZ-PRS or PE-PRS), the AD-PRS and the interaction factor between psychiatric PRS and AD-PRS, at the 10 different P_T_ listed above. Moreover, the first 10 genetic PCs were used as covariates in the regression models to control for any potential effects of population stratification. Since psychoses mainly occur at an advanced disease stage, the same models were re-run including also the MMSE as an additional covariate to control for disease severity. The significance threshold was set at *p* < 0.05. Goodness of fit of the logistic regression models was determined by means of C statistics, a measure equivalent to the Area Under the Receiver Operating Characteristic curve [AUROC; 48].

A secondary logistic regression analysis was run to test the association between the AD-PRS and AD diagnosis.

### MRI data and pre-processing

The structural T1-weighted MRI scan collected at the time-point closest to the neuropsychiatric assessment was selected for each participant. All MRI data were acquired as specified in the ADNI MRI protocol [49] at either 1.5 T (*n* = 248) or 3 T (*n* = 552). Pooling of MRI data acquired at different MR field strengths has been previously shown to be a valid approach with small effects on neurovolumetric quantifications [50–52]. The steps of the standard voxel-based morphometry (VBM) protocol [53] were carried out with Matlab (Mathworks Inc., UK) and Statistical Parametric Mapping (SPM) 12 (Wellcome Centre for Human Neuroimaging, London, UK): 1) images were reoriented to the bi-commissural axis; 2) reoriented images were segmented to separate 3 tissues, i.e. GM, white matter and cerebro-spinal fluid; 3) GM maps were modulated and then registered to the standard International Consortium of Brain Mapping (ICBM) template in the MNI space; and finally, 4) normalised images were smoothed with an 8 mm full-width at half-maximum Gaussian kernel. The global volume of each tissue map was quantified using SPM12 and, finally, the total intracranial volume (TIV) was calculated for each participant by summing the values of all 3 extracted tissues.

An ANOVA was used to compare GM volumes across groups and three *t*-tests were implemented for pair-wise post hoc comparisons (HC vs AD-NP, HC vs AD-PS and AD-PS vs AD-NP). Multiple regression models were created to test the association between SCZ-PRS and PE-PRS (at all P_T_) and GM volume in the whole sample (n = 800) and in the three groups independently (HC, AD-PS and AD-NP). The first 10 genetic PCs, age, education, sex, magnetic field strength, TIV and GM ratio (i.e. GM volume/TIV) and testing site were included as covariates in all VBM models. The same regression analyses were run to assess also the association between AD-PRS and regional GM volume. The cluster-forming significance threshold was set at *p* < 0.001 and results were corrected for multiple comparisons at cluster level (*p* < 0.05 FWE-corrected).

Subsequently, post hoc exploratory analyses were carried out on twenty-six GM regions of interest (ROIs), 13 ROIs in each hemisphere, selected on the basis of the VBM results reflecting the association between the SCZ-PRS and GM volume in the AD-PS group, to investigate 1) volumetric differences between patient groups with MANCOVA models and 2) associations between ROI volumes and psychosis severity in the AD-PS group with multiple regression. The Automated Anatomical Labelling (AAL) atlas 2 [54] was used to extract ROI volumes of: hippocampus, amygdala, parahippocampal gyrus, rectus gyrus, medial prefrontal cortex, middle and superior frontal gyri (orbital parts), middle and superior temporal gyri, fusiform gyrus, globus pallidus, middle and inferior occipital gyri. The same covariates included in the VBM models were used and Bonferroni correction for multiple comparisons was applied to the significance threshold (*p* < 0.0019).

### Statistical analyses

Demographic and clinical characteristics were compared across groups using ANOVA for continuous variables, with Bonferroni correction for post hoc tests, and *χ*^2^ for categorical variables.

All analyses were carried out using *R* (www.r-project.org/) and robust standard errors were estimated to calculate 95% confidence intervals of odds ratios (ORs) resulting from logistic regressions [55] (see also: sandwich.r-forge.r-project.org/). VBM analyses were carried out using Statistical Parametric Mapping 12 (Wellcome Centre for Human Neuroimaging, London, UK).

## Results

### Clinical profile

The AD-PS group was significantly older than both HC and AD-NP groups and had fewer years of education than the HC group, although differences were only marginally significant (Table 1). Differences in sex distributions were also found across groups: the AD-PS group included more men than the other groups. Significant associations were found between *APOE* ε4 genotype and both diagnosis and psychosis, since the proportion of ε4 carriers was higher in the AD-PS than in the AD-NP group (χ^2^ = 16.04, *p* < 0.001).

**Table 1:** Demographic and clinical profiles (means ± SD) of the three participant groups

Variables	AD-PS (*n* = 121)	AD-NP (*n* = 476)	HC (*n* = 203)	F	*p*
Age (years)	77.07 ± 7.83	73.51 ± 7.99	73.86 ± 5.79	11.18	0.16 × 10^–4^
Education (years)	15.70 ± 2.78	16.06 ± 2.80	16.51 ± 2.61	3.57	0.029
Sex (F/M)^a^	54/67	198/278	106/97	6.49^b^	0.039
*APOE* ε4 ^a, c^	77/44	206/270	49/154	50.25^b^	1.28 × 10^–11^
NPI-Q total score	8.14 ± 4.85	1.89 ± 2.51	0.35 ± 0.87	331.45	1.78 × 10^–5^
MMSE total score	22.46 ± 5.87	27.21 ± 2.83	29.06 ± 1.17	160.23	3.89 × 10^–59^
CDT (Drawing)	3.44 ± 1.52	4.43 ± 0.89	4.74 ± 0.50	71.31	3.51 × 10^–29^
CDT (Copy)	4.18 ± 1.27	4.73 ± 0.59	4.88 ± 0.34	39.08	6.62 × 10^–17^
LMT (Immediate)	5.39 ± 4.26	9.33 ± 4.08	14.67 ± 2.93	236.07	4.85 × 10^–81^
LMT (Delayed)	3.13 ± 4.13	7.00 ± 4.13	13.68 ± 3.16	314.51	5.18 × 10^–101^
CFT (Animals)	12.83 ± 6.14	17.25 ± 5.51	21.25 ± 5.58	83.31	1.54 × 10^–33^
TMT-A (seconds)	63.82 ± 43.04	41.00 ± 21.08	33.22 ± 10.38	61.62	1.40 × 10^–25^
TIV (ml)	1468.39 ± 161.54	1455.42 ± 141.36	1433.17 ± 137.25	2.67	0.070
GM ratio	0.38 ± 0.05	0.41 ± 0.05	0.43 ± 0.04	41.94	4.79 × 10^–18^

*APOE* Apolipoprotein E, *CDT* Clock Drawing Test, *GM* Grey matter, *LMT* Logical Memory Test, *MMSE* Mini Mental State Examination, *MRF* Magnetic Resonance Field, *NPI-Q* Neuropsychiatric Inventory Questionnaire, *TIV* Total Intracranial Volume, *TMT-A* Trail Making Test—part A

^a^Frequencies

^b^χ^2^

^c^Carriers/non-carriers

Overall, both patient groups showed more severe neuropsychiatric symptoms and worse cognitive performance than the HC group. However, the AD-PS group had a more severe clinical profile than the AD-NP group characterised by higher NPI-Q scores (Table 2), worse cognitive deficits and lower GM ratio (i.e. less GM tissue available in proportion to head size). In fact, only 17.4% of patients in the AD-NP group had dementia, compared to 59.5% of the patients in the AD-PS group.

**Table 2:** Comparison of frequencies of neuropsychiatric symptoms across patient groups (χ^2^, *p* < 0.05)

Neuropsychiatric symptoms	AD-PS (*n* = 121)	AD-NP (*n* = 476)	χ^2^	*p*
Delusions	89 (73.6%)	0 (0%)	411.46	1.76 × 10^–91^
Hallucinations	44 (36.4%)	0 (0%)	186.86	1.54 × 10^–42^
Agitation	65 (53.7%)	69 (14.5%)	85.26	2.61 × 10^–20^
Depression	57 (47.1%)	117 (24.6%)	23.71	0.01 × 10^–4^
Anxiety	61 (50.4%)	56 (11.8%)	91.46	1.14 × 10^–21^
Euphoria	8 (6.6%)	11 (2.3%)	5.79	0.016
Apathy	65 (53.7%)	85 (17.9%)	65.95	4.16 × 10^–16^
Disinhibition	48 (39.7%)	43 (9.0%)	70.09	5.68 × 10^–17^
Irritability	73 (60.3%)	120 (25.2%)	54.39	1.64 × 10^–13^
Motor disturbance	35 (28.9%)	25 (5.3%)	59.81	1.05 × 10^–14^
Sleep problems	46 (38.0%)	97 (20.4%)	16.48	0.49 × 10^–4^
Appetite problems	55 (45.5%	52 (10.9%)	78.20	9.33 × 10^–19^

### Association between PRSs and psychosis status

All logistic regression models significantly predicted psychosis in AD, but only the AD-PRS was significantly associated with psychosis across all P_T_ (Table 3). When the analyses were run including the MMSE, this variable was the only one significantly associated with psychotic status across patients. The value of the C statistics was about 0.65 for all models that included only PRSs, while it increased to about 0.81 when the MMSE was entered as a covariate.

**Table 3:** Associations (odds ratios—ORs) between PRSs (both SCZ-PRS and PE-PRS) and psychosis status in patients with AD

P_T_	Predictor	Psychosis (only PRSs)				Psychosis (PRSs + MMSE)			
		OR	95% CI	*p*	C	OR	95% CI	*p*	C
*Schizophrenia*									
5 × 10^–8^	SCZ-PRS	0.98	0.78–1.22	0.83	0.65	1.00	0.78–1.28	0.97	0.81
	AD-PRS	**1.34**	**1.10–1.63**	**4.15 × 10**^**–3**^		1.09	0.86–1.39	0.48	
	SCZ × AD	1.00	0.81–1.22	0.97		1.07	0.86–1.33	0.81	
	MMSE	–-	–-	–-		**0.75**	**0.70–0.81**	**5.30 × 10**^**–14**^	
1 × 10^–6^ 1 × 10^–5^	SCZ-PRS	0.97	0.77–1.22	0.77	0.65	0.99	0.76–1.24	0.80	0.81
	AD-PRS	**1.35**	**1.11–1.65**	**3.11 × 10**^**–3**^		1.10	0.86–1.40	0.46	
	SCZ × AD	1.02	0.82–1.26	0.86		1.13	0.90–1.40	0.29	
	MMSE	–-	–-	–-		**0.75**	**0.70–0.81**	**5.34 × 10**^**–14**^	
	SCZ-PRS	0.99	0.78–1.26	0.93	0.65	1.01	0.77–1.32	0.96	0.81
	AD-PRS	**1.36**	**1.12–1.67**	**2.30 × 10**^**–3**^		1.11	0.87–1.41	0.40	
	SCZ × AD	0.97	0.78–1.21	0.81		1.03	0.82–1.29	0.78	
	MMSE	–-	–-	–-		**0.75**	**0.70–0.81**	**5.93 × 10**^**–14**^	
0.0001	SCZ-PRS	0.98	0.76–1.25	0.85	0.65	1.00	0.76–1.32	0.99	0.81
	AD-PRS	**1.37**	**1.12–1.67**	**2.44 × 10**^**–3**^		1.11	0.87–1.41	0.41	
	SCZ × AD	1.03	0.83–1.27	0.79		1.10	0.87–1.38	0.42	
	MMSE	–-	–-	–-		**0.75**	**0.70–0.81**	**3.66 × 10**^**–14**^	
0.001	SCZ-PRS	0.92	0.71–1.20	0.55	0.66	0.95	0.71–1.27	0.73	0.81
	AD-PRS	**1.38**	**1.12–1.69**	**2.15 × 10**^**–3**^		1.12	0.88–1.42	0.37	
	SCZ × AD	1.07	0.86–1.33	0.52		1.11	0.88–1.40	0.37	
	MMSE	–-	–-	–-		**0.75**	**0.70–0.81**	**5.38 × 10**^**–14**^	
0.01	SCZ-PRS	0.90	0.65–1.27	0.56	0.66	1.01	0.69–1.49	0.95	0.81
	AD-PRS	**1.39**	**1.13–1.70**	**2.03 × 10**^**–3**^		1.12	0.88–1.42	0.35	
	SCZ × AD	1.09	0.85–1.40	0.50		1.12	0.85–1.48	0.44	
	MMSE	–-	–-	–-		**0.75**	**0.70–0.81**	**4.23 × 10**^**–14**^	
0.05	SCZ-PRS	0.94	0.64–1.37	0.73	0.65	1.01	0.65–1.56	0.98	0.81
	AD-PRS	**1.39**	**1.12–1.71**	**2.24 × 10**^**–3**^		1.11	0.87–1.42	0.38	
	SCZ × AD	1.08	0.83–1.40	0.57		1.14	0.84–1.55	0.38	
	MMSE	–-	–-	–-		**0.75**	**0.70–0.81**	**3.93 × 10**^**–14**^	
0.1	SCZ-PRS	0.92	0.61–1.39	0.69	0.65	0.97	0.61–1.56	0.92	0.81
	AD-PRS	**1.39**	**1.12–1.71**	**2.42 × 10**^**–3**^		1.11	0.87–1.42	0.39	
	SCZ × AD	1.07	0.81–1.41	0.62		1.15	0.83–1.58	0.40	
	MMSE	–-	–-	–-		**0.75**	**0.70–0.81**	**4.81 × 10**^**–14**^	
0.5	SCZ-PRS	0.75	0.48–1.18	0.22	0.66	0.79	0.46–1.37	0.41	0.81
	AD-PRS	**1.41**	**1.13–1.75**	**1.93 × 10**^**–3**^		1.13	0.88–1.45	0.34	
	SCZ × AD	1.10	0.81–1.48	0.54		1.17	0.82–1.67	0.38	
	MMSE	–-	–-	–-		**0.75**	**070–0.81**	**9.23 × 10**^**–14**^	
1	SCZ-PRS	0.77	0.48–1.24	0.28	0.66	0.81	0.45–1.46	0.49	0.81
	AD-PRS	**1.42**	**1.14–1.76**	**1.81 × 10**^**–3**^		1.14	0.88–1.47	0.32	
	SCZ × AD	1.14	0.83–1.56	0.42		1.21	0.83–1.76	0.31	
	MMSE	–-	–-	–-		**0.75**	**070–0.81**	**8.51 × 10**^**–14**^	
*Psychotic experiences*									
1 × 10^–5^	PE-PRS	1.03	0.83–1.30	0.77	0.66	0.95	0.73–1.15	0.72	0.80
	AD-PRS	**1.39**	**1.13–1.70**	**1.76 × 10**^**–3**^		1.11	0.88–1.41	0.37	
	PE × AD	0.88	0.71–1.09	0.24		0.98	0.77–1.25	0.87	
	MMSE	–-	–-	–-		**0.75**	**0.70–0.81**	**7.47 × 10**^**–14**^	
0.0001	PE-PRS	1.04	0.84–1.28	0.73	0.66	1.00	0.78–1.28	1.00	0.81
	AD-PRS	**1.39**	**1.13–1.71**	**1.87 × 10**^**–3**^		1.11	0.87–1.41	0.40	
	PE × AD	0.90	0.73–1.10	0.31		0.98	0.77–1.25	0.87	
	MMSE	–-	–-	–-		**0.75**	**0.70–0.81**	**7.75 × 10**^**–14**^	
0.001	PE-PRS	1.17	0.94–1.46	0.15	0.66	1.13	0.87–1.46	0.36	0.81
	AD-PRS	**1.37**	**1.12–1.69**	**2.77 × 10**^**–3**^		1.12	0.88–1.43	0.36	
	PE × AD	0.96	0.78–1.17	0.69		0.94	0.73–1.22	0.65	
	MMSE	–-	–-	–-		**0.76**	**0.70–0.81**	**1.68 × 10**^**–13**^	
0.01	PE-PRS	1.01	0.81–1.27	0.90	0.66	0.98	0.75–1.27	0.87	0.81
	AD-PRS	**1.39**	**1.13–1.71**	**1.95 × 10**^**–3**^		1.12	0.88–1.43	0.34	
	PE × AD	0.92	0.74–1.15	0.47		0.93	0.72–1.20	0.56	
	MMSE	–-	–-	–-		**0.75**	**0.70–0.81**	**1.28 × 10**^**–13**^	
0.05	PE-PRS	1.03	0.82–1.30	0.78	0.65	1.02	0.78–1.32	0.90	0.81
	AD-PRS	**1.37**	**1.12–1.69**	**2.80 × 10**^**–3**^		1.10	0.87–1.40	0.43	
	PE × AD	0.99	0.79–1.24	0.94		0.97	0.75–1.25	0.80	
	MMSE	–-	–-	–-		**0.75**	**0.70–0.81**	**8.91 × 10**^**–14**^	
0.1	PE-PRS	1.03	0.82–1.29	0.79	0.65	1.00	0.77–1.28	0.98	0.80
	AD-PRS	**1.38**	**1.12–1.70**	**2.38 × 10**^**–3**^		1.10	0.87–1.41	0.43	
	PE × AD	0.94	0.75–1.18	0.60		0.95	0.74–1.21	0.67	
	MMSE	–-	–-	–-		**0.75**	**0.70–0.81**	**9.21 × 10**^**–14**^	
0.5	PE-PRS	0.96	0.77–1.21	0.75	0.65	0.93	0.72–1.19	0.56	0.80
	AD-PRS	**1.38**	**1.12–1.70**	**2.10 × 10**^**–3**^		1.10	0.86–1.41	0.44	
	PE × AD	0.96	0.77–1.20	0.74		0.98	0.77–1.25	0.86	
	MMSE	–-	–-	–-		**0.75**	**0.70–0.81**	**1.01 × 10**^**–13**^	
1	PE-PRS	0.95	0.75–1.19	0.65	0.65	0.89	0.69–1.15	0.39	0.81
	AD-PRS	**1.38**	**1.12–1.70**	**2.18 × 10**^**–3**^		1.10	0.86–1.41	0.44	
	PE × AD	0.99	0.79–1.23	0.91		1.01	0.79–1.29	0.93	
	MMSE	–-	–-	–-		**0.75**	**0.70–0.81**	**7.65 × 10**^**–14**^	

*AD* Alzheimer’s Disease, *MMSE* Mini Mental State Examination, *PE* Psychotic Experiences, *PRS* Polygenic Risk Score, *SCZ* Schizophrenia

Significant results are reported in bold text

Logistic regression analysis on diagnosis prediction showed that the AD-PRS was significantly associated with higher risk of AD at all P_T_ (Supplementary Information – Table S1).

### Association between PRSs and GM volume

**Fig. 1:**
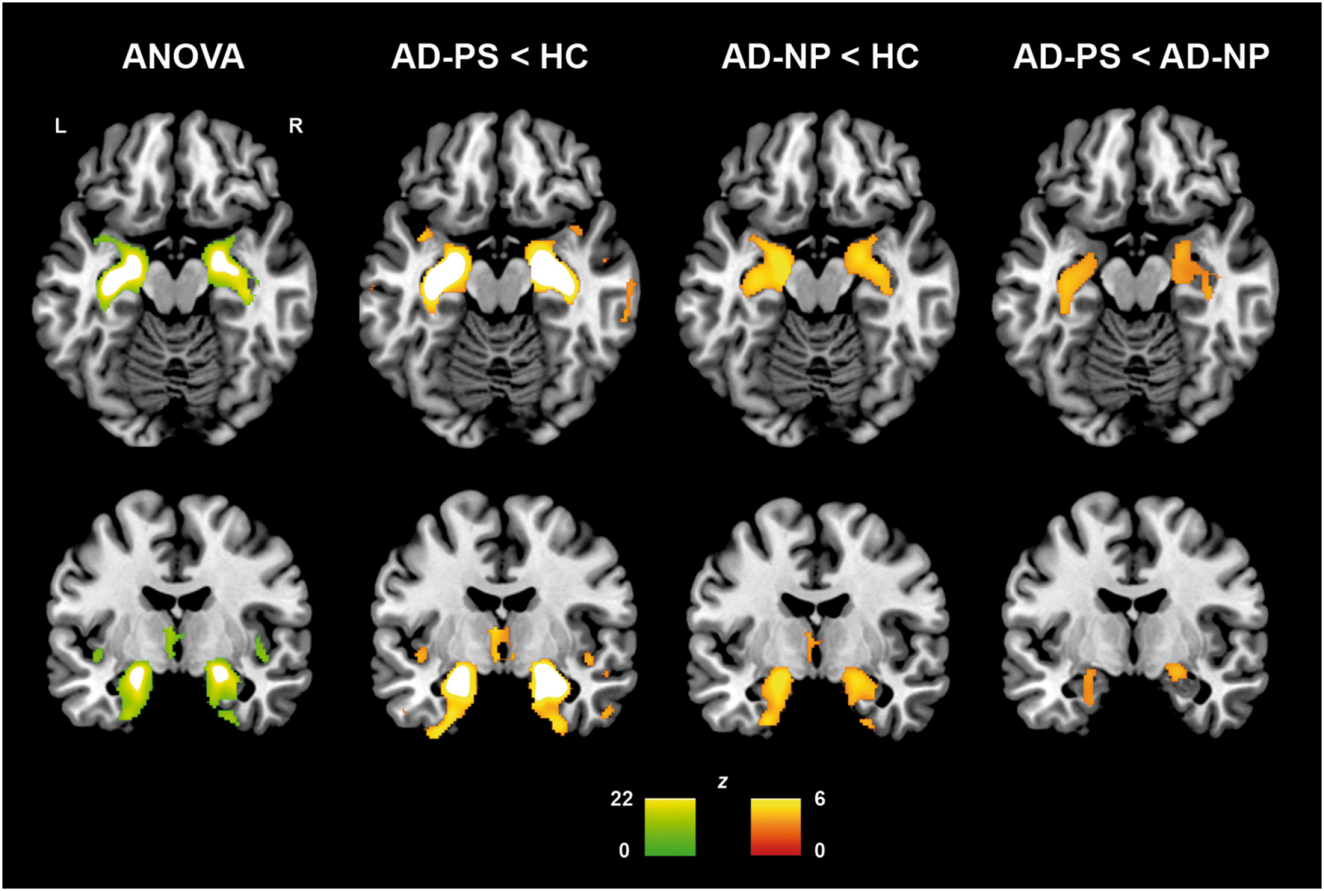
GM volume differences (independent-sample *t*-tests) between groups of participants (cluster-level FWE-corrected *p* = 0.05)

VBM ANOVA analysis showed significant differences across groups in bilateral medio-temporal areas. As expected, post hoc independent-sample *t*-tests found that both patients groups showed bilateral GM atrophy in medio-temporal areas when compared with the HC group (Fig. 1). Moreover, atrophy was significantly more severe in the AD-PS than in the AD-NP group (peak coordinates in Supplementary Information—Table S2).

**Fig. 2:**
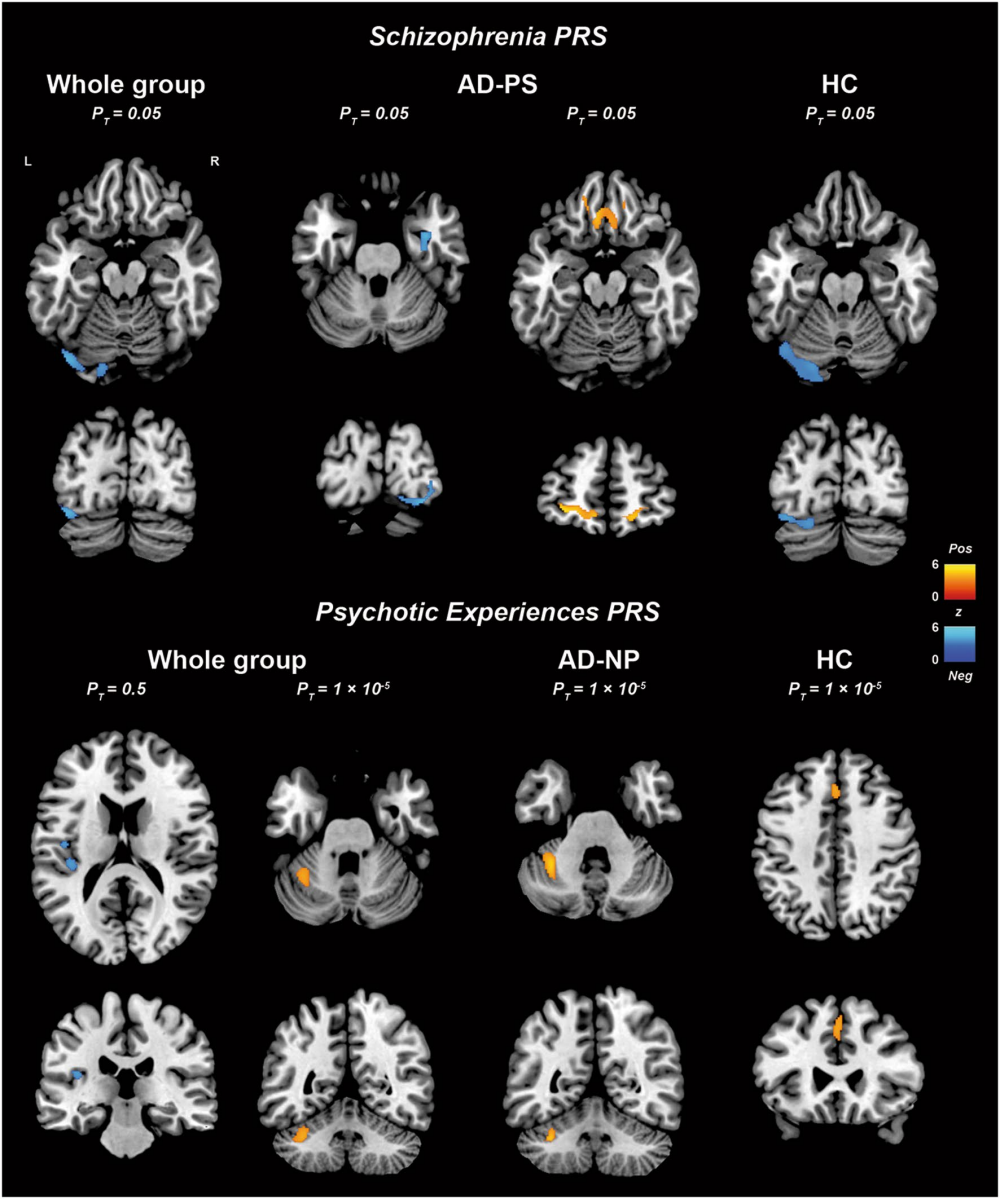
Results of the multiple regression analysis showing the negative (blue) and positive (red) associations between psychiatric PRSs (SCZ-PRS and PE-PRS) and GM volume in the whole sample and in sub-groups (cluster-level FWE-corrected *p* = 0.05)

VBM regression analyses found that higher SCZ-PRS values were associated mainly, but inconsistently across P_T_, with smaller volume in occipital areas in the whole sample and in the HC group (Fig. 2) (Supplementary Information—Table S3 and Figure S1). In the AD-PS groups, instead, the SCZ-PRS was positively associated with the volume of bilateral medial prefrontal (mPFC) and orbitofrontal cortices (OFC) and negatively with the volume of right-lateralised medio-temporal and basal ganglia GM areas, as well as some occipital clusters, but only at less conservative thresholds (P_T_ = 0.05 and P_T_ = 0.1) (Fig. 2 and Table 4; for a comprehensive representation of all results see Supplementary Information—Figure S2). No significant genetic-neurovolumetric associations were observed for the AD-NP group.

**Table 4:** Associations between the SCZ-PRS and GM regional volumes in the AD-PS group (cluster-level FWE-corrected *p* < 0.05)

P_T_	Cluster extent	Side	Brain region	*t* value	MNI coordinates		
					*x*	*y*	*z*
*Positive association*							
**0.01**	493	L	Medial PFC (BA 10)	4.38	-28	54	-8
		L	Medial PFC (BA 10)	4.34	-9	60	-4
		L	Medial PFC (BA 10)	4.04	-16	63	6
**0.05**	1545	L	MFG (BA 10)	5.22	-30	52	-6
		L	SFG (BA 10)	4.91	-10	60	-4
		L	SFG (BA 10)	4.72	-18	63	8
		L	RG (BA 11)	4.67	-4	32	-20
		R	SFG (BA 11)	4.62	18	54	-10
		L	OFC (BA 11)	4.42	-6	36	-18
**0.1**	961	L	MFG (BA 11)	4.86	-28	52	-8
		L	RG (BA 11)	4.62	-4	32	-20
		L	SFG (BA 10)	4.49	-10	60	-4
*Negative association*							
**5 × 10**^**–8**^	986	R	FG (BA 20)	4.79	36	-6	-22
		R	Lateral GP	4.60	22	-14	-8
		R	STG (BA 22)	3.63	40	-24	-12
**1 × 10**^**–6**^	1211	R	Hippocampus	4.98	34	-8	-24
		R	Lateral GP	4.54	22	-14	-8
		R	PHG (BA 26)	4.07	40	-26	-12
**1 × 10**^**–5**^	1202	R	Hippocampus	5.17	33	-12	-27
		R	Lateral GP	4.35	22	-15	-8
		R	PHG (BA 26)	4.24	40	-26	-12
**0.0001**	1128	R	Hippocampus	5.11	33	-14	-27
		R	Lateral GP	4.85	22	-15	-8
		R	PHG (BA 26)	4.47	40	-26	-12
**0.001**	1510	R	Hippocampus	5.21	33	-14	-27
		R	Lateral GP	4.54	22	-14	-9
		R	Hippocampus	3.67	33	-27	-15
**0.01**	910	R	Hippocampus	4.94	33	-15	-26
		R	Hippocampus	4.20	34	-4	-27
		R	Lateral GP	4.09	26	-12	-10
**0.05** **0.1**	789	R	IOG (BA 18)	4.56	30	-90	-15
		R	FG (BA 19)	4.24	44	-75	-21
	497	R	MTG (BA 21)	4.44	36	-4	-26
		R	Hippocampus	3.94	33	-14	-27
		R	Amygdala	3.84	26	-10	-10
	729	R	IOG (BA 18)	4.42	28	-92	-14
		R	MOG (BA 18)	4.14	34	-90	-4
		R	FG (BA 19)	4.06	44	-75	-21

*BA* Brodmann area, *FG* Fusiform Gyrus, *GP* Globus Pallidus, *IOG* Inferior Occipital Gyrus, *MFG* Middle Frontal Gyrus, *MOG* Middle Occipital Gyrus, *MTG* Middle Temporal Gyrus, *OFC* Orbitofrontal Cortex, *PHG* Parahippocampal Gyrus, *PFC* Prefrontal Cortex, *RG* Rectus Gyrus, *SFG* Superior Frontal Gyrus, *STG* Superior Temporal Gyrus

More inconsistent associations were observed between the PE-PRS and regional GM volumes (Supplementary Information—Table S3). In the whole sample of participants, the PE-PRS was negatively associated with the volume of left-lateralised insulo-temporal cortices and positively with the volume of a cerebellar cluster. However, the PE-PRS was positively associated with GM volume in cerebellar and occipital areas in the AD-NP group, and in cingulate and medial frontal areas in the HC group. No significant results were found for patients with psychosis.

VBM regression models investigating the effect of the AD-PRS on GM volume showed significant negative associations with clusters in bilateral medio-temporal and right inferior temporal and posterior cingulate areas, in the whole sample, and in left-lateralised medio-temporal areas, in the AD-PS group (Supplementary Information—Table S4 and Figure S3).

When volumes of GM ROIs (selected on the basis of the findings of whole brain analyses) were compared between patient groups, bilateral medial and right middle temporal areas were the only ones to be significantly more atrophic in patients with psychosis than in those without (Table 5). Severity of psychosis in the AD-PS group was not associated with any ROI volumes (Supplementary Information—Table S5).

**Table 5:** Comparisons of GM volumes (means ± SD) of twenty-six ROIs between patient groups (Bonferroni-corrected *p* < 0.0019)

ROIs	AD-PS (*n* = 121)	AD-NP (*n* = 476)	*F*	*p*
Left hippocampus	**3.60 ± 0.50**	**3.89 ± 0.47**	**20.75**	**6.38 × 10**^**–6**^
Right hippocampus	**3.37 ± 0.51**	**3.70 ± 0.48**	**26.11**	**4.36 × 10**^**–7**^
Left PHG	**3.41 ± 0.51**	**3.73 ± 0.48**	**24.06**	**1.21 × 10**^**–6**^
Right PHG	**4.07 ± 0.58**	**4.38 ± 0.54**	**17.93**	**2.65 × 10**^**–5**^
Left amygdala	**0.85 ± 0.14**	**0.94 ± 0.13**	**22.36**	**2.83 × 10**^**–6**^
Right amygdala	**0.91 ± 0.13**	**0.99 ± 0.13**	**21.68**	**3.97 × 10**^**–6**^
Left MTG	14.24 ± 2.19	15.25 ± 2.00	5.06	0.025
Rigt MTG	**13.00 ± 2.11**	**14.05 ± 1.82**	**14.80**	**1.33 × 10**^**–4**^
Left STG	6.07 ± 1.05	6.58 ± 1.01	0.97	0.324
Right STG	8.12 ± 1.47	8.93 ± 1.32	9.41	0.002
Left rectus gyrus	2.49 ± 0.43	2.60 ± 0.43	0.88	0.349
Right rectus gyrus	2.29 ± 0.38	2.39 ± 0.37	0.54	0.462
Left mPFC	1.89 ± 0.31	1.98 ± 0.33	0.81	0.368
Right mPFC	2.47 ± 0.39	2.58 ± 0.41	0.80	0.372
Left MFG	2.48 ± 0.37	2.57 ± 0.40	1.24	0.265
Rigt MFG	2.69 ± 0.48	2.80 ± 0.49	0.56	0.455
Left SFG	2.48 ± 0.38	2.57 ± 0.41	0.64	0.425
Right SFG	2.62 ± 0.39	2.73 ± 0.40	0.46	0.497
Left globus pallidus	0.47 ± 0.10	0.49 ± 0.09	0.58	0.445
Right globus pallidus	0.45 ± 0.10	0.48 ± 0.10	1.72	0.190
Left fusiform gyrus	8.51 ± 1.24	9.06 ± 1.12	2.42	0.120
Right fusiform gyrus	10.73 ± 1.39	11.27 ± 1.26	2.68	0.102
Left IOG	2.66 ± 0.47	2.84 ± 0.40	0.04	0.843
Right IOG	2.40 ± 0.46	2.62 ± 0.40	1.54	0.215
Left MOG	8.26 ± 1.21	8.79 ± 1.21	0.01	0.915
Right MOG	5.31 ± 1.01	5.71 ± 0.82	0.06	0.807

*IOG* Inferior Occipital Gyrus, *MFG* Middle Frontal Gyrus, *MOG* Middle Occipital Gyrus, *mPFC* Medial Prefrontal Cortex, *MTG* Middle Temporal Gyrus, *PHG* Parahippocampal Gyrus, *ROI* Region of Interest, *SFG* Superior Frontal Gyrus, *STG* Superior Temporal Gyrus

Significant results surviving statistical correction for multiple comparisons are reported in bold text

## Discussion

Genetic analyses revealed that only the AD-PRS, among all those investigated, was significantly associated with psychosis status in this sample of patients with AD, when disease severity was not accounted for. However, all the models only yielded C statistics values < 0.80, thus indicating that currently PRSs alone may have limited utility for prediction of this phenotype in a clinical setting. On the contrary, when the MMSE was included among the predictors, it emerged as the only variable significantly associated with psychosis and led to a considerable increase in model fit (C > 0.80). Associations between PRSs and neurovolumetric features, instead, were not univocal, although more consistent between the SCZ-PRS and GM volume in medio-temporal and OFC/mPFC areas in the AD-PS group.

The lack of associations between the SCZ-PRS and the clinical psychotic phenotype in our sample is in contrast with significant, although inconsistent, findings from previous investigations. Indeed, DeMichele-Sweet et al. [25] observed that psychotic symptoms in AD were associated with a set of genes but inversely with a SCZ-PRS that appeared to be protective against psychosis in their cohort. However, a recent comprehensive meta-analysis found a significant positive association between a SCZ-PRS and risk of psychosis in AD by combining 11 cohorts of patients [26]. Nevertheless, quite inconsistent results emerged in individual cohorts and across P_T_ investigated, probably due to either limited power or differences in age, MMSE score and gender proportions across cohorts. It must be noted that the ADNI sample selected by Creese et al. [26] differs from ours as it appears smaller and the reported confidence intervals are only partially overlapping with ours. Other methodological differences may have also contributed to the divergence in results, namely: the GWAS summary statistics used to compute the SCZ-PRS, the number of genetic PCs accounted for in the analyses and the inclusion of the AD-PRS and interaction factors in our logistic regression models.

No significant results emerged for the PE-PRS, a metric that should capture non-specific risk for any type of self-reported psychotic experiences in the general population and that is associated with a range of disorders [43]. These findings may be due to the low phenotypic variance explained by the PE-PRS and, therefore, no association with AD-related psychoses are likely to be detected if these symptoms are mainly driven by a set of more disease-specific genes as proposed by DeMichele-Sweet et al. [25].

Overall, it appears that the risk of psychosis in AD may be primarily linked to genetic liability for AD, consistently with previous evidence suggesting that the *APOE* ε4 allele (potentially with some additional contribution of other AD risk genes) is the main fosterer of psychosis in this population [15–17]. In fact, the AD-PS group comprised a significantly higher proportion of ε4 carriers than the AD-NP group. After controlling for disease severity, however, this association did not survive, hence suggesting that psychotic symptoms may be more prevalent either in advanced disease stages [2] or in more severe cases with higher AD-PRS and more pronounced neurodegeneration. Consistently with this hypothesis, between-group comparisons highlighted worse cognitive decline and medio-temporal lobe atrophy for the AD-PS compared to the AD-NP group.

VBM regression analyses on the PE-PRS showed largely inconsistent positive associations with GM volume of cerebellar/occipital areas, at a conservative P_T_, in the whole sample and in the AD-PS group and with GM volume in the anterior cingulate in the HC group. The PE-PRS was also associated with lower GM volume in left insular/superior temporal areas in the whole sample, in line with previous findings in the general population [56] and in patients with AD [57]. However, no significant associations were detected in the AD-PS group, hence suggesting that PE-PRS may not be a contributing factor for AD-related psychoses.

The SCZ-PRS, instead, was negatively associated with the volume in occipital regions in the whole sample and in the HC group, and even in the AD-PS group at some less conservative P_T_. The relevance of these findings is unclear because of the inconsistency observed across the tested PRS thresholds and evidence from previous observations of a positive association between SCZ-PRS and occipital GM volume [58]. In the AD-PS group, the SCZ-PRS was associated with more reliable neural signatures, largely dissociable from those detected for the AD-PRS. While the AD-PRS was associated with lower GM volume in left-lateralised temporal areas, the SCZ-PRS was associated with larger volume in OFC/mPFC areas, involved in reward processing and social behaviours, and lower volume in right-sided medio-temporal areas, crucial for memory and emotion processing, and in the right globus pallidus, involved in motor control and executive functions. The negative associations with medio-temporal and basal ganglia volumes are in line with previous investigations on SCZ patients [59, 60], although these findings were not replicated in all studies [61, 62]. Moreover, previous observations had already shown that altered volumetric features of the OFC may be implicated in SCZ onset [63, 64].

The SCZ-PRS exerted opposite effects on medio-temporal/subcortical and frontal areas and this appears to be consistent with previous accounts [65] of GM loss and disruption in functional connectivity between medio-temporal and OFC areas associated with behavioural disturbance in a mouse model of SCZ [22]. The same pattern of alteration in hippocampus-OFC functional connectivity was also found associated with SCZ-PRS in people with SCZ and their unaffected first-degree relatives [66]. Moreover, altered functional connectivity in frontal areas including the OFC has also been observed in patients with AD and delusions [10]. Therefore, it appears that a dysfunction in the communication between medio-temporal and orbitofrontal areas, the volumes of which were associated with SCZ-PRS in the AD-PS group, may play a central role in the manifestation of psychotic symptoms also in patients with AD.

It must be noted that SCZ, differently from AD, is commonly regarded as a neurodevelopmental disorder [67] influenced by interacting genetic and environmental factors that often lead to disease onset in late adolescence/early adulthood [68, 69]. To the best of our knowledge, participants included in this study presented with psychotic symptoms only in older adulthood, after substantial AD-related pathological changes had occurred. Therefore, psychotic symptoms in AD may manifest when neurodegeneration exceeds a certain threshold in a cluster of brain areas in susceptible individuals at greater genetic risk. Psychosis may, therefore, emerge when a pattern of latent neural vulnerability in certain brain regions, shaped by polygenic risk for SCZ, is unveiled by the progression of neurodegeneration. In fact, the comparison between patient groups also showed preserved GM volume in frontal areas that was positively associated with the SCZ-PRS in the AD-PS group. Hence, patients with AD and psychosis appear to be those presenting with a neurovolumetric pattern suggestive of a disconnection between medio-temporal and OFC areas similar to that observed in SCZ [66], i.e. a combination of severe medio-temporal atrophy, mainly due to AD pathology but potentially also influenced by SCZ risk, and preserved volume in OFC/mPFC areas associated with higher polygenic risk for SCZ.

Some limitations to this work must be mentioned. First, the AD-PS group has been defined pooling all types of psychotic symptoms, but it is likely that individual symptoms may be characterised by partially distinct aetiologies, neural and neuropathological correlates [3, 70, 71]. Second, the limited sample size, especially for the HC and AD-PS groups. Third, the inclusion of 52 participants from a minority ethnic background might have introduced variability in the data not related to the phenotype of interest; however, we decided to retain these datasets in the analyses to maximise the power of this study and increase its diversity, and included 10 genetic PCs in all regression models to minimise any bias. Fourth, we only included MRI data on brain macrostructure (those available almost universally across ADNI participants) while we did not investigate functional brain MRI data; indeed altered brain activity may represent, compared with GM atrophy, a predominant neural process associated with psychosis in AD. Finally, previously it has been suggested that the SCZ-PRS (though this could in principle be extended to any PRS derived from a complex trait) is a heterogeneous construct that captures the effect of many biological pathways associated with the disorder [72]. As such, there is some evidence that restricting SCZ-PRS to sets of functionally related genes might improve its predictive capabilities in the context of neuroimaging studies [60]. However, we did not attempt these complex partitioning procedures since, to date, no large gene sets that could result in a well-powered PRS have been associated specifically with psychotic experiences.

In summary, this study provides novel findings that suggest that polygenic risk for SCZ is associated with a specific neural configuration in patients with AD and psychoses. In particular, the combination of smaller medio-temporal volumes but preserved OFC/mPFC volumes may signal a disconnection between these two systems, already implicated in SCZ, and foster the neural conditions that generate a cluster of behavioural alterations that could explain psychotic presentations in people with AD. Future investigations combining genetic as well as brain activity/metabolism data will be better placed to clarify the role of the medio-temporal-orbitofrontal disconnection in the genesis of psychoses in AD.

## Supplementary Information

Below is the link to the electronic supplementary material.

Supplementary file1 (DOCX 1301 kb)

## Data Availability

All ADNI data are made publicly available.

## Footnotes

1 The ADNI was launched in 2003 as a public–private partnership, led by Principal Investigator Michael W. Weiner, MD. The primary goal of ADNI has been to test whether serial magnetic resonance imaging (MRI), positron emission tomography (PET), other biological markers, and clinical and neuropsychological assessment can be combined to measure the progression of mild cognitive impairment (MCI) and early Alzheimer’s disease (AD). For up-to-date information, see www.adni-info.org.
