# Supplementary material for: The neural signatures of psychoses in Alzheimer’s disease: a neuroimaging genetics approach
Source: Eur Arch Psychiatry Clin Neurosci. 2022 Jun 21;273(1):253–67. doi: 10.1007/s00406-022-01432-6 (PMC9957843; doi:10.1007/s00406-022-01432-6)
Supplement: Supplementary file 1 — Supplementary file1 (DOCX 1301 kb) [file 406_2022_1432_MOESM1_ESM.docx]

**Table S1.** Association (odds rations - OR) between AD-PRS and AD diagnosis.

| **P_T_** | **OR** | **95% CI** | ***p*** | **C** |
| --- | --- | --- | --- | --- |
| **5 × 10^-8^** | 1.67 | 1.40-1.99 | 1.58 × 10^-8^ | 0.65 |
| **1 × 10^-6^** | 1.68 | 1.40-2.00 | 1.11 × 10^-8^ | 0.65 |
| **1 × 10^-5^** | 1.69 | 1.41-2.02 | 0.85 × 10^-8^ | 0.65 |
| **0.0001** | 1.70 | 1.42-2.03 | 0.59 × 10^-8^ | 0.65 |
| **0.001** | 1.70 | 1.42-2.03 | 0.65 × 10^-8^ | 0.65 |
| **0.01** | 1.72 | 1.44-2.06 | 0.27 × 10^-8^ | 0.65 |
| **0.05** | 1.78 | 1.49-2.13 | 0.05 × 10^-8^ | 0.66 |
| **0.1** | 1.79 | 1.49-2.15 | 0.04 × 10^-8^ | 0.66 |
| **0.5** | 1.84 | 1.53-2.22 | 0.01 × 10^-8^ | 0.67 |
| **1** | 1.85 | 1.54-2.23 | 0.01 × 10^-8^ | 0.67 |

**Table S2.** Results of the ANOVA and *post hoc* pair-wise comparisons (*t*-tests) of GM maps (FWE-corrected cluster-level *p* < 0.05).

| **Analysis** | **Cluster extent** | **Side** | **Brain region** | **Statistic value** | **MNI coordinates** | | |
| --- | --- | --- | --- | --- | --- | --- | --- |
|  |  |  |  |  | ***x*** | ***y*** | ***z*** |
| **ANOVA** |  |  |  | ***F*** |  |  |  |
|  | 5837 | L | Hippocampus | 29.97 | -33 | -24 | -10 |
|  |  | L | Hippocampus | 28.86 | -30 | -14 | -20 |
|  |  | L | Amygdala | 27.89 | -21 | -8 | -15 |
|  |  | L | Amygdala | 27.52 | -27 | 2 | -21 |
|  |  | L | Uncus (BA 36) | 22.02 | -20 | 0 | -32 |
|  |  | L | Uncus (BA 28) | 17.97 | -27 | -10 | -36 |
|  |  | L | Thalamus | 15.42 | -3 | -9 | 8 |
|  |  | L | Thalamus (medial dorsal nucleus) | 15.35 | -4 | -14 | 9 |
|  |  | L | Thalamus | 14.74 | -4 | -3 | 0 |
|  | 3957 | R | Amygdala | 28.29 | 22 | -6 | -15 |
|  |  | R | Hippocampus | 26.66 | 33 | -26 | -10 |
|  |  | R | Hippocampus | 26.00 | 30 | -10 | -20 |
|  |  | R | Amygdala | 22.74 | 24 | 2 | -20 |
|  |  | R | Uncus (BA 28) | 18.49 | 18 | 0 | -28 |
|  |  | R | Uncus (BA 28) | 17.74 | 20 | 3 | -34 |
|  |  | R | Uncus (BA 36) | 13.95 | 27 | -8 | -38 |
|  | 418 | R | STG (BA 22) | 11.27 | 45 | -9 | -6 |
|  |  | R | STG (BA 22) | 9.66 | 51 | -18 | -8 |
|  |  | R | STG (BA 22) | 7.87 | 50 | -30 | 0 |
| ***Post hoc*** |  |  |  | ***t*** |  |  |  |
| **HC > AD-PS** | 6014 | L | Hippocampus | 8.49 | -26 | -12 | -16 |
|  |  | L | Hippocampus | 7.76 | -32 | -24 | -12 |
|  |  | L | Hippocampus | 7.34 | -28 | -32 | -8 |
|  | 6986 | R | Amygdala | 8.31 | 22 | -8 | -15 |
|  |  | R | Hippocampus | 7.48 | 33 | -24 | -10 |
|  |  | R | Uncus (BA 28) | 6.60 | 20 | 4 | -34 |
|  | 1689 | L | Thalamus | 5.17 | -4 | -8 | 9 |
|  |  | L | Thalamus | 5.08 | -4 | -2 | 0 |
|  |  | L | Thalamus | 4.81 | 4 | 2 | 0 |
|  | 431 | L | ITG (BA 20) | 4.90 | -58 | -21 | -28 |
|  |  | L | ITG (BA 20) | 4.66 | -51 | -14 | -34 |
|  | 487 | R | STG (BA 22) | 4.80 | 52 | -18 | -6 |
| **HC > AD-NP** | 5185 | L | Amygdala | 5.88 | -27 | 2 | -21 |
|  |  | L | Amygdala | 5.78 | -20 | -8 | -15 |
|  |  | L | Uncus (BA 28) | 5.61 | -20 | 3 | -32 |
|  | 2842 | R | Hippocampus | 5.37 | 30 | -10 | -20 |
|  |  | R | Amygdala | 5.28 | 22 | 0 | -21 |
|  |  | R | Hippocampus | 4.58 | 34 | -26 | -12 |
| **AD-NP > AD-PS** | 2349 | L | PHG (BA 36) | 4.92 | -36 | -33 | -12 |
|  |  | L | Hippocampus | 4.76 | -33 | -21 | -12 |
|  |  | L | Hippocampus | 4.66 | -28 | -30 | -9 |
|  | 2024 | R | Hippocampus | 4.83 | 32 | -27 | -8 |
|  |  | R | Amygdala | 4.56 | 22 | -4 | -14 |
|  |  | R | PHG (BA 36) | 4.50 | 40 | -26 | -10 |

BA: Brodmann area, ITG: Inferior temporal gyrus, PHG: Parahippocampal gyrus, STG: Superior temporal gyrus

**Table S3.** Associations between the SCZ-PRS and PE-PRS and GM regional volumes in the whole group of participants, the AD-NP and the HC groups (FWE-corrected cluster-level *p* < 0.05).

| **Group** | **P_T_** | **Cluster extent** | **Side** | **Brain region** | ***t* value** | **MNI coordinates** | | |
| --- | --- | --- | --- | --- | --- | --- | --- | --- |
|  |  |  |  |  |  | ***x*** | ***y*** | ***z*** |
|  | *SCZ-PRS - Negative association* | | | | | | | |
| ***Whole group*** | **0.01** | 431 | L | FG (BA 19) | 4.31 | -40 | -78 | -16 |
|  |  |  | L | FG (BA 18) | 4.29 | -18 | -88 | -21 |
|  |  |  | L | IOG (BA 18) | 4.23 | -30 | -86 | -21 |
|  | **0.05** | 461 | L | FG (BA 19) | 4.41 | -40 | -78 | -16 |
|  |  |  | L | FG (BA 18) | 4.00 | -18 | -88 | -21 |
|  |  |  | L | IOG (BA 18) | 3.99 | -30 | -86 | -21 |
| ***HC*** | **0.05** | 1023 | L | FG (BA 19) | 3.99 | -42 | -75 | -16 |
|  |  |  | L | FG (BA 19) | 3.72 | -22 | -80 | -21 |
|  |  |  | L | FG (BA 18) | 3.68 | -20 | -87 | -20 |
|  | **0.1** | 901 | L | FG (BA 19) | 4.35 | -40 | -75 | -16 |
|  |  |  | L | FG (BA 18) | 3.92 | -33 | -82 | -20 |
|  |  |  | L | FG (BA 19) | 3.69 | -20 | -87 | -20 |
|  | **0.5** | 542 | L | MFG (BA 8) | 5.15 | -38 | 15 | 46 |
|  |  |  | L | MFG (BA 6) | 4.79 | -48 | 0 | 39 |
|  |  |  | L | MFG (BA 8) | 4.36 | -42 | 6 | 46 |
|  |  | 687 | L | FG (BA 19) | 4.14 | -39 | -76 | -18 |
|  |  |  | L | LG (BA 18) | 4.03 | -18 | -86 | -20 |
|  | *PE-PRS - Negative association* | | | | | | | |
| ***Whole group*** | **0.5** | 386 | L | Insula (BA 13) | 4.10 | -46 | -10 | 14 |
|  |  |  | L | Insula (BA 13) | 4.00 | -39 | -27 | 18 |
|  |  |  | L | STG (BA 22) | 3.80 | -46 | -10 | 4 |
|  | **1** | 408 | L | Insula (BA 13) | 4.19 | -45 | -10 | 14 |
|  |  |  | L | STG (BA 22) | 3.96 | -46 | -10 | 4 |
|  |  |  | L | Insula (BA 13) | 3.88 | -39 | -28 | 18 |
|  | *PE-PRS - Positive association* | | | | | | | |
| ***Whole group*** | **1 × 10^-5^** | 511 | L | Culmen | 4.06 | -34 | -54 | -32 |
|  |  |  | L | Uvula | 3.28 | -27 | -66 | -32 |
|  | **0.001** | 399 | R | ISL | 4.36 | 18 | -74 | -46 |
| ***AD-NP*** | **1 × 10^-5^** | 740 | R | Cuneus (BA 19) | 5.19 | 8 | -84 | 27 |
|  |  |  | R | Cuneus (BA 17) | 4.04 | 16 | -81 | 2 |
|  |  |  | R | Cuneus (BA 18) | 3.60 | 10 | -92 | 8 |
|  |  | 410 | L | Culmen | 4.80 | -33 | -50 | -33 |
|  | **0.001** | 439 | L | LG (BA 18) | 4.66 | -26 | -96 | -10 |
|  |  |  | L | MOG (BA 18) | 3.64 | -18 | -100 | 12 |
| ***HC*** | **1 × 10^-5^** | 508 | L | ACC (BA 32) | 4.03 | -2 | 33 | 33 |
|  |  |  | R | Medial SFG (BA 8) | 3.93 | 0 | 26 | 44 |

ACC: Anterior cingulate cortex, BA: Brodmann area, FG: Fusiform gyrus, , IOG: Inferior occipital gyrus, ISL: Inferior semilunar lobule, LG: Lingual gyrus, MFG: Middle frontal gyrus, MOG: Middle occipital gyrus, SFG: Superior frontal gyrus, STG: Superior temporal gyrus

**Table S4.** Associations between the AD-PRS and GM regional volumes in the whole group of participants and in the AD-PS group (FWE-corrected cluster-level *p* < 0.05).

| **Group** | **P_T_** | **Cluster extent** | **Side** | **Brain region** | ***t* value** | **MNI coordinates** | | |
| --- | --- | --- | --- | --- | --- | --- | --- | --- |
|  |  |  |  |  |  | ***x*** | ***y*** | ***z*** |
|  | *Negative association* | | | | | | | |
| ***Whole group*** | **5 × 10^-8^** | 2533 | R | Hippocampus | 5.58 | 30 | -33 | -4 |
|  |  |  | R | Hippocampus | 5.14 | 30 | -10 | -21 |
|  |  |  | R | Amygdala | 5.04 | 21 | -8 | -16 |
|  |  | 2416 | L | Hippocampus | 5.61 | -27 | -14 | -18 |
|  |  |  | L | Uncus (BA 28) | 4.33 | -16 | -3 | -28 |
|  |  |  | L | Hippocampus | 4.18 | -27 | -32 | -8 |
|  |  | 436 | R | PCC (BA 31) | 4.43 | 3 | -44 | 32 |
|  |  |  | R | Precuneus (BA 31) | 3.71 | 3 | -54 | 30 |
|  |  |  | R | Precuneus (BA 7) | 3.42 | 2 | -63 | 42 |
|  |  | 609 | R | FG (BA 20) | 4.43 | 42 | -16 | -30 |
|  |  |  | R | FG (BA 37) | 4.35 | 50 | -48 | -20 |
|  |  |  | R | FG (BA 20) | 4.27 | 46 | -27 | -24 |
|  | **1 × 10^-6^** | 2602 | R | Hippocampus | 5.67 | 28 | -33 | -4 |
|  |  |  | R | Hippocampus | 5.20 | 30 | -10 | -21 |
|  |  |  | R | Amygdala | 5.06 | 21 | -8 | -15 |
|  |  | 2540 | L | Hippocampus | 5.72 | -27 | -14 | -18 |
|  |  |  | L | Uncus (BA 28) | 4.48 | -16 | -3 | -28 |
|  |  |  | L | Hippocampus | 4.25 | -27 | -32 | -8 |
|  |  | 456 | R | PCC (BA 31) | 4.48 | 3 | -44 | 32 |
|  |  |  | R | Precuneus (BA 31) | 3.75 | 3 | -54 | 30 |
|  |  |  | R | Precuneus (BA 7) | 3.43 | 2 | -62 | 42 |
|  |  | 628 | R | FG (BA 20) | 4.47 | 42 | -16 | -30 |
|  |  |  | R | FG (BA 37) | 4.34 | 50 | -48 | -20 |
|  |  |  | R | FG (BA 20) | 4.28 | 46 | -27 | -24 |
|  | **1 × 10^-5^** | 2656 | R | Hippocampus | 5.69 | 28 | -33 | -4 |
|  |  |  | R | Hippocampus | 5.18 | 30 | -10 | -21 |
|  |  |  | R | Amygdala | 5.09 | 21 | -8 | -15 |
|  |  | 2585 | L | Hippocampus | 5.65 | -27 | -14 | -18 |
|  |  |  | L | Uncus (BA 28) | 4.49 | -16 | -3 | -28 |
|  |  |  | L | Hippocampus | 4.30 | -26 | -36 | -6 |
|  |  | 433 | R | PCC (BA 31) | 4.48 | 3 | -42 | 32 |
|  |  |  | R | Precuneus (BA 31) | 3.66 | 3 | -54 | 30 |
|  |  |  | R | Precuneus (BA 7) | 3.39 | 2 | -62 | 42 |
|  |  | 633 | R | FG (BA 20) | 4.57 | 42 | -16 | -30 |
|  |  |  | R | FG (BA 37) | 4.35 | 48 | -50 | -20 |
|  |  |  | R | FG (BA 20) | 4.26 | 45 | -26 | -26 |
|  | **0.0001** | 2906 | R | Hippocampus | 6.11 | 28 | -33 | -4 |
|  |  |  | R | Hippocampus | 5.46 | 30 | -10 | -21 |
|  |  |  | R | Amygdala | 5.39 | 21 | -8 | -15 |
|  |  | 2766 | L | Hippocampus | 5.76 | -27 | -14 | -18 |
|  |  |  | L | Uncus (BA 28) | 4.60 | -16 | -3 | -28 |
|  |  |  | L | Hippocampus | 4.43 | -26 | -33 | -4 |
|  |  | 624 | R | FG (BA 20) | 4.68 | 42 | -16 | -30 |
|  |  |  | R | FG (BA 20) | 4.34 | 45 | -26 | -26 |
|  |  |  | R | FG (BA 37) | 4.33 | 48 | -50 | -20 |
|  |  | 412 | R | PCC (BA 31) | 4.43 | 3 | -44 | 32 |
|  |  |  | R | Precuneus (BA 31) | 3.66 | 3 | -54 | 30 |
|  |  |  | R | Precuneus (BA 7) | 3.37 | 2 | -62 | 40 |
|  | **0.001** | 2682 | R | Hippocampus | 5.73 | 28 | -33 | -4 |
|  |  |  | R | Hippocampus | 5.25 | 30 | -10 | -21 |
|  |  |  | R | Amygdala | 5.12 | 21 | -8 | -16 |
|  |  | 2666 | L | Hippocampus | 5.78 | -27 | -14 | -18 |
|  |  |  | L | Uncus (BA 28) | 4.62 | -16 | -3 | -28 |
|  |  |  | L | Hippocampus | 4.45 | -26 | -33 | -6 |
|  |  | 562 | R | FG (BA 20) | 4.79 | 42 | -16 | -30 |
|  |  |  | R | FG (BA 37) | 4.40 | 48 | -50 | -20 |
|  |  |  | R | FG (BA 20) | 4.38 | 44 | -26 | -26 |
|  |  | 382 | R | PCC (BA 31) | 4.80 | 3 | -44 | 32 |
|  |  |  | R | Precuneus (BA 31) | 3.95 | 3 | -54 | 30 |
|  | **0.01** | 2770 | R | Hippocampus | 5.69 | 28 | -33 | -4 |
|  |  |  | R | Hippocampus | 5.36 | 30 | -10 | -21 |
|  |  |  | R | Amygdala | 5.18 | 21 | -8 | -16 |
|  |  | 2764 | L | Hippocampus | 5.86 | -26 | -14 | -20 |
|  |  |  | L | Uncus (BA 28) | 4.85 | -16 | -3 | -28 |
|  |  |  | L | Hippocampus | 4.50 | -26 | -33 | -6 |
|  |  | 488 | R | FG (BA 20) | 5.02 | 42 | -16 | -30 |
|  |  |  | R | FG (BA 20) | 4.56 | 44 | -26 | -26 |
|  | **0.05** | 2942 | L | Hippocampus | 6.07 | -24 | -14 | -20 |
|  |  |  | L | Uncus (BA 28) | 4.97 | -16 | -3 | -28 |
|  |  |  | L | Hippocampus | 4.64 | -26 | -33 | -6 |
|  |  | 2915 | R | Hippocampus | 5.84 | 28 | -33 | -4 |
|  |  |  | R | Hippocampus | 5.42 | 30 | -10 | -21 |
|  |  |  | R | Amygdala | 5.34 | 21 | -6 | -16 |
|  |  | 538 | R | FG (BA 20) | 5.14 | 42 | -16 | -30 |
|  |  |  | R | FG (BA 20) | 4.68 | 44 | -26 | -26 |
|  | **0.1** | 2938 | R | Hippocampus | 5.89 | 28 | -33 | -4 |
|  |  |  | R | Hippocampus | 5.41 | 30 | -10 | -21 |
|  |  |  | R | Amygdala | 5.37 | 21 | -6 | -16 |
|  |  | 2990 | L | Hippocampus | 6.09 | -24 | -14 | -20 |
|  |  |  | L | Uncus (BA 28) | 5.00 | -16 | -3 | -28 |
|  |  |  | L | Hippocampus | 4.70 | -26 | -33 | -6 |
|  |  | 565 | R | FG (BA 20) | 5.21 | 42 | -18 | -30 |
|  | **0.5** | 2920 | R | Hippocampus | 5.94 | 28 | -33 | -4 |
|  |  |  | R | Amygdala | 5.31 | 21 | -6 | -16 |
|  |  |  | R | Hippocampus | 5.29 | 30 | -10 | -21 |
|  |  | 2995 | L | Hippocampus | 5.99 | -26 | -14 | -20 |
|  |  |  | L | Uncus (BA 36) | 4.74 | -21 | -4 | -36 |
|  |  |  | L | Hippocampus | 4.61 | -26 | -33 | -6 |
|  |  | 569 | R | FG (BA 20) | 5.33 | 42 | -16 | -30 |
|  | **1** | 2946 | R | Hippocampus | 5.97 | 28 | -33 | -4 |
|  |  |  | R | Hippocampus | 5.36 | 30 | -10 | -21 |
|  |  |  | R | Amygdala | 5.34 | 21 | -8 | -16 |
|  |  | 3006 | L | Uncus (BA 28) | 5.97 | -26 | -14 | -20 |
|  |  |  | L | Hippocampus | 4.59 | -26 | -33 | -6 |
|  |  | 591 | R | FG (BA 20) | 5.36 | 42 | -16 | -30 |
| ***AD-PS*** | **1 × 10^-6^** | 421 | L | Amygdala | 4.61 | -18 | -6 | -10 |
|  |  |  | L | PHG (BA 34) | 3.87 | -15 | -2 | -18 |
|  | **1 × 10^-5^** | 450 | L | Amygdala | 4.66 | -18 | -6 | -10 |
|  |  |  | L | PHG (BA 34) | 3.92 | -15 | -2 | -20 |
|  | **0.0001** | 444 | L | Amygdala | 4.68 | -18 | -6 | -10 |
|  |  |  | L | PHG (BA 34) | 3.91 | -15 | -2 | -20 |
|  | **0.001** | 401 | L | Amygdala | 4.56 | -18 | -6 | -10 |
|  |  |  | L | PHG (BA 34) | 3.81 | -15 | -2 | -18 |
|  | **0.01** | 465 | L | Amygdala | 4.61 | -18 | -6 | -10 |
|  |  |  | L | PHG (BA 34) | 3.93 | -15 | -2 | -20 |
|  | **0.05** | 477 | L | Amygdala | 4.53 | -20 | -8 | -12 |
|  |  |  | L | PHG (BA 34) | 3.92 | -15 | -2 | -20 |
|  | **0.1** | 487 | L | Amygdala | 4.57 | -20 | -8 | -12 |
|  |  |  | L | PHG (BA 34) | 3.85 | -15 | -2 | -20 |
|  | **0.5** | 396 | L | Amygdala | 4.22 | -18 | -6 | -10 |
|  |  |  | L | PHG (BA 34) | 3.67 | -15 | -2 | -20 |

BA: Brodmann area, FG: Fusiform gyrus, PCC: Posterior cingulate cortex, PHG: Parahippocampal gyrus

**Table S5.** Associations between volumes of GM ROIs and severity of psychosis in the AD-PS group.

| **ROIs** | **β** | ***p*** |
| --- | --- | --- |
| **Left hippocampus** | -0.022 | 0.896 |
| **Right hippocampus** | 0.011 | 0.948 |
| **Left parahippocampal gyrus** | -0.010 | 0.950 |
| **Right parahippocampal gyrus** | 0.013 | 0.946 |
| **Left amygdala** | <0.001 | 0.997 |
| **Right amygdala** | -0.060 | 0.707 |
| **Left middle temporal gyrus** | -0.143 | 0.573 |
| **Rigt middle temporal gyrus** | 0.093 | 0.730 |
| **Left superior temporal gyrus** | -0.227 | 0.283 |
| **Right superior temporal gyrus** | -0.198 | 0.403 |
| **Left rectus gyrus** | 0.018 | 0.895 |
| **Right rectus gyrus** | -0.039 | 0.781 |
| **Left medial prefrontal cortex** | -0.085 | 0.557 |
| **Right medial prefrontal cortex** | -0.120 | 0.453 |
| **Left middle frontal gyrus** | -0.105 | 0.515 |
| **Rigt middle frontal gyrus** | -0.114 | 0.392 |
| **Left superior frontal gyrus** | -0.020 | 0.890 |
| **Right superior frontal gyrus** | -0.037 | 0.818 |
| **Left globus pallidus** | -0.029 | 0.792 |
| **Right globus pallidus** | -0.040 | 0.719 |
| **Left fusiform gyrus** | -0.121 | 0.655 |
| **Right fusiform gyrus** | -0.297 | 0.280 |
| **Left inferior occipital gyrus** | 0.004 | 0.981 |
| **Right inferior occipital gyrus** | 0.012 | 0.944 |
| **Left middle occipital gyrus** | 0.034 | 0.865 |
| **Right middle occipital gyrus** | 0.191 | 0.314 |

**
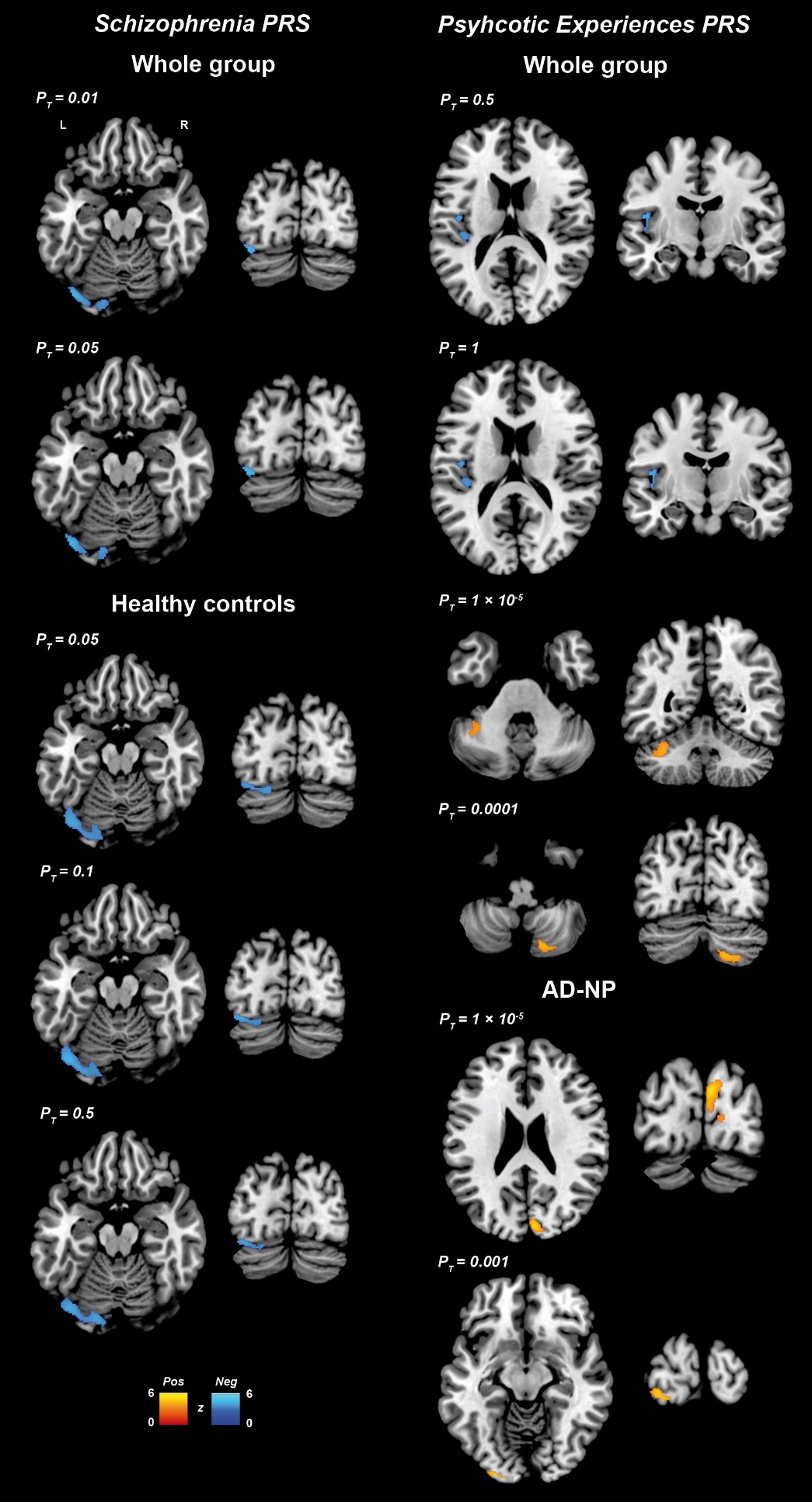
**

**Figure S1.** Results of the multiple regression analysis showing negative (blue) and positive (red) associations between psychiatric PRSs (SCZ-PRS and PE-PRS) and GM volume in the whole sample and across groups at all P_T_ (FWE-corrected cluster-level *p* = 0.05)


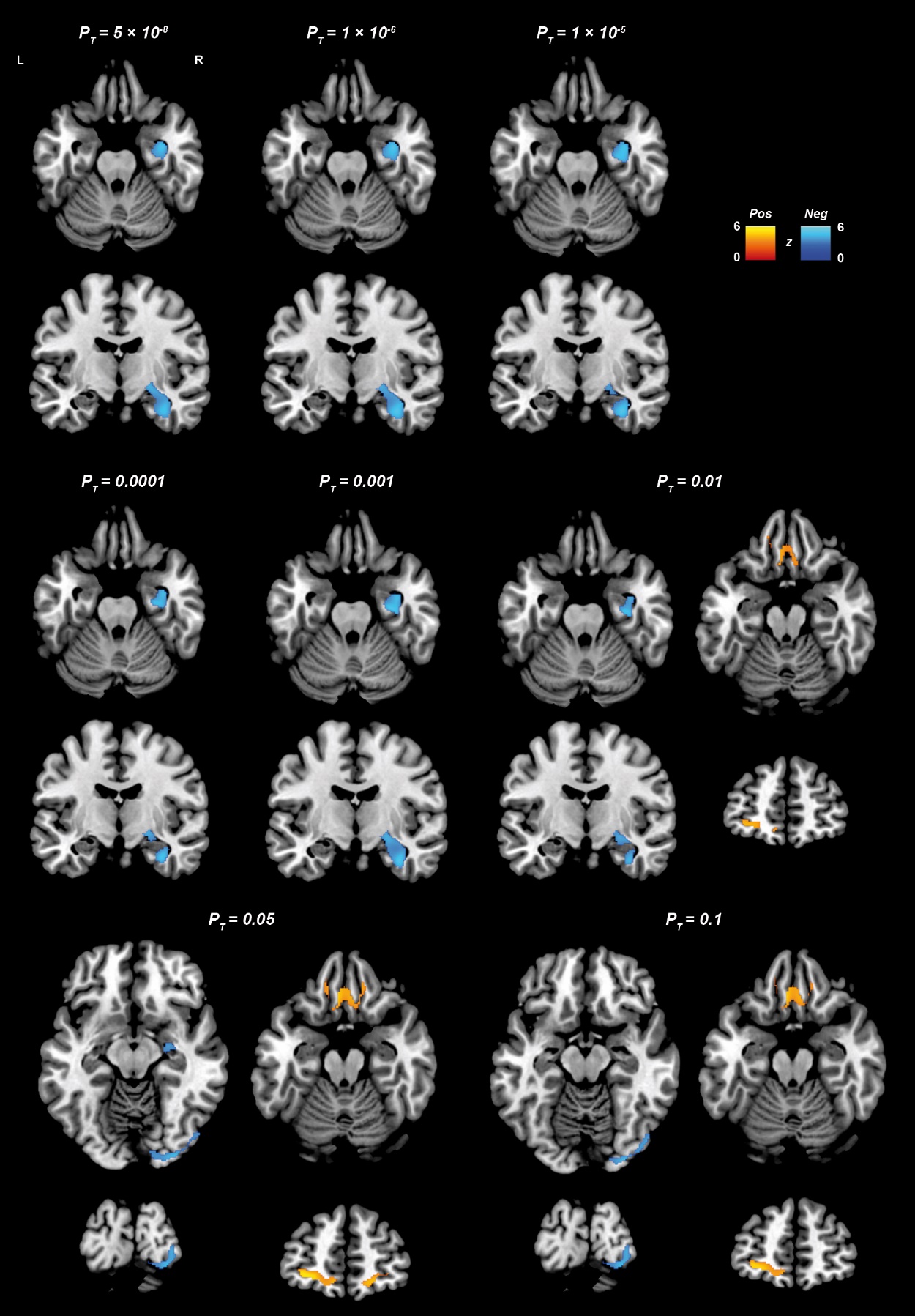


**Figure S2.** Results of the multiple regression analysis showing negative (blue) and positive (red) associations between the SCZ-PRS and GM volume in the AD-PS group at all P_T_ (FWE-corrected cluster-level *p* = 0.05)


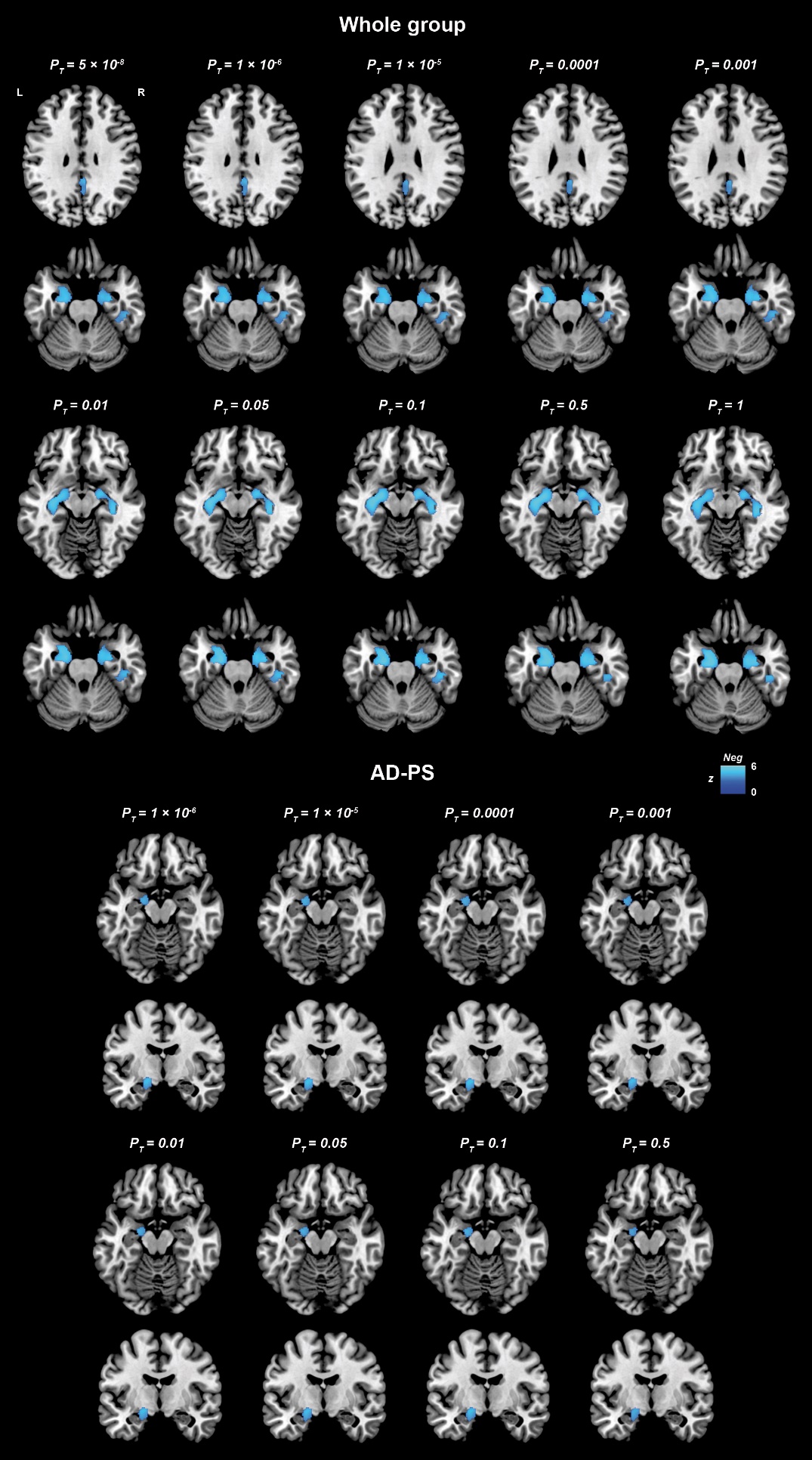


**Figure S3.** Results of the multiple regression analysis showing negative associations between the AD-PRS and GM volume in the whole sample and in the AD-PS group at all P_T_ (FWE-corrected cluster-level *p* = 0.05)
